# Application of metal stable isotopes labeling and elemental mass spectrometry for biomacromolecule profiling

**DOI:** 10.52601/bpr.2024.240039

**Published:** 2025-04-30

**Authors:** Ping Zhang, Ying Han, Yue Xu, Liang Gao

**Affiliations:** 1 Department of Chemistry, College of Chemistry and Life Science, Beijing University of Technology, Beijing 100124, China

**Keywords:** Biomacromolecule, Metal stable isotope labeling, Mass spectrometry analysis

## Abstract

Biomacromolecules including proteins and nucleic acids are widely recognized for their pivotal and irreplaceable role in maintaining the normal functions of biological systems. By combining metal stable isotope labeling with elemental mass spectrometry, researchers can quantify the amount and track the spatial distribution of specific biomacromolecules in complex biological systems. In this review, the probes classification and metal stable isotope labeling strategies are initially summarized. Secondly, the technical characteristics and working principle of the elemental mass spectrometry techniques including inductively coupled plasma mass spectrometry and secondary ion mass spectrometry are introduced to achieve highly sensitive detection of multiple biomacromolecules at molecular, cellular and tissue levels. Lastly, we underline the advantages and limitations of elemental mass spectrometry combined with metal stable isotope labeling strategies, and propose the perspectives for future developments.

## INTRODUCTION

Biomacromolecules, including proteins and nucleic acids, perform diverse physiological activities throughout an organism’s life cycle (Sun *et al*. 2021; Zhang *et al*. 2018b). Changes in the abundance and distribution of these biomacromolecules can serve as indicators of disease occurrence and therapeutic outcomes (Karimzadeh *et al*. 2020). Therefore, analysis of the abundance and spatial distribution of vital biomacromolecules is essential for clinical diagnosis and pharmacological evaluation (Lv *et al*. 2013; Sun *et al*. 2020).

Mass spectrometry (MS), invented by Francis William Aston in 1919, has evolved into a widely used analytical technique for investigating small molecules and biomacromolecules over the past century. The underlying principle involves ionizing the sample by an ion source to generate charged ions, which are subsequently accelerated by an electric field to form an ion beam directed into a mass analyzer. Within this analyzer, ions with different mass-to-charge ratios experience opposite velocity dispersion due to the combined effects of electric and magnetic fields, ultimately resulting in the generation of a mass spectrum that provides information about their respective masses. With advancements in ion source technologies, various branches of mass spectrometry have emerged including electrospray ionization mass spectrometry (ESI-MS), matrix-assisted laser desorption/ionization mass spectrometry (MALDI-MS), inductively coupled plasma mass spectrometry (ICP-MS), secondary ion mass spectrometry (SIMS), among others.

The analytical methods for biomacromolecules by MS can be normally classified into label-free and labeled approaches. Label-free detection of biomacromolecules can be realized by molecular MS, such as ESI-MS and MALDI-MS. Following sample preparation and separation, biomolecule identification can be accomplished through molecular MS combined with peptide sequencing via tandem MS (MS/MS), as well as through database-driven protein identification using bioinformatics (Cristoni and Bernardi 2003).

The classic label-based approaches for analyzing biomacromolecules refer to widely accepted immunoassay methods. For instance, immunofluorescence imaging and flow cytometry analysis are widely employed in biomacromolecule analysis due to the advantages of simplicity, high sensitivity, and rapid response (Sargazi *et al*. 2022). Unfortunately, multiple detection is limited by the overlap of emission bandwidth and the quenching of fluorescent dyes. Therefore, it is a great challenge to simultaneously measure the biomacromolecules with the abundance differing in an order of magnitude, or simultaneously measuring kinds of targets (Anyaegbu *et al*. 2019; Hulspas *et al*. 2009). Additionally, immunofluorescence imaging is susceptibly interfered with by the autofluorescence and scattering light derived from samples, lacking reliable quantification procedures (Chen *et al*. 2009; Liu *et al*. 2016).

Metal stable isotope labeling, coupled with highly sensitive elemental MS technology, such as ICP-MS and SIMS offers a compelling alternative to fluorescence detection and has proven effective in quantifying various target molecules (Liu *et al*. 2014). By replacing fluorescent groups with stable metal isotopes for antibody or ligand labeling, the abundance of each isotope can be correlated with specific antibody probes, allowing for precise measurement of antigen levels. In theory, ICP-MS and SIMS provide high sensitivity and isotopic resolution for over 100 elements, highlighting the great potential of metal stable isotope labeling for advanced multi-dimensional analysis (Han *et al*. 2013; Zhang *et al*. 2020a).

**Figure 1:**
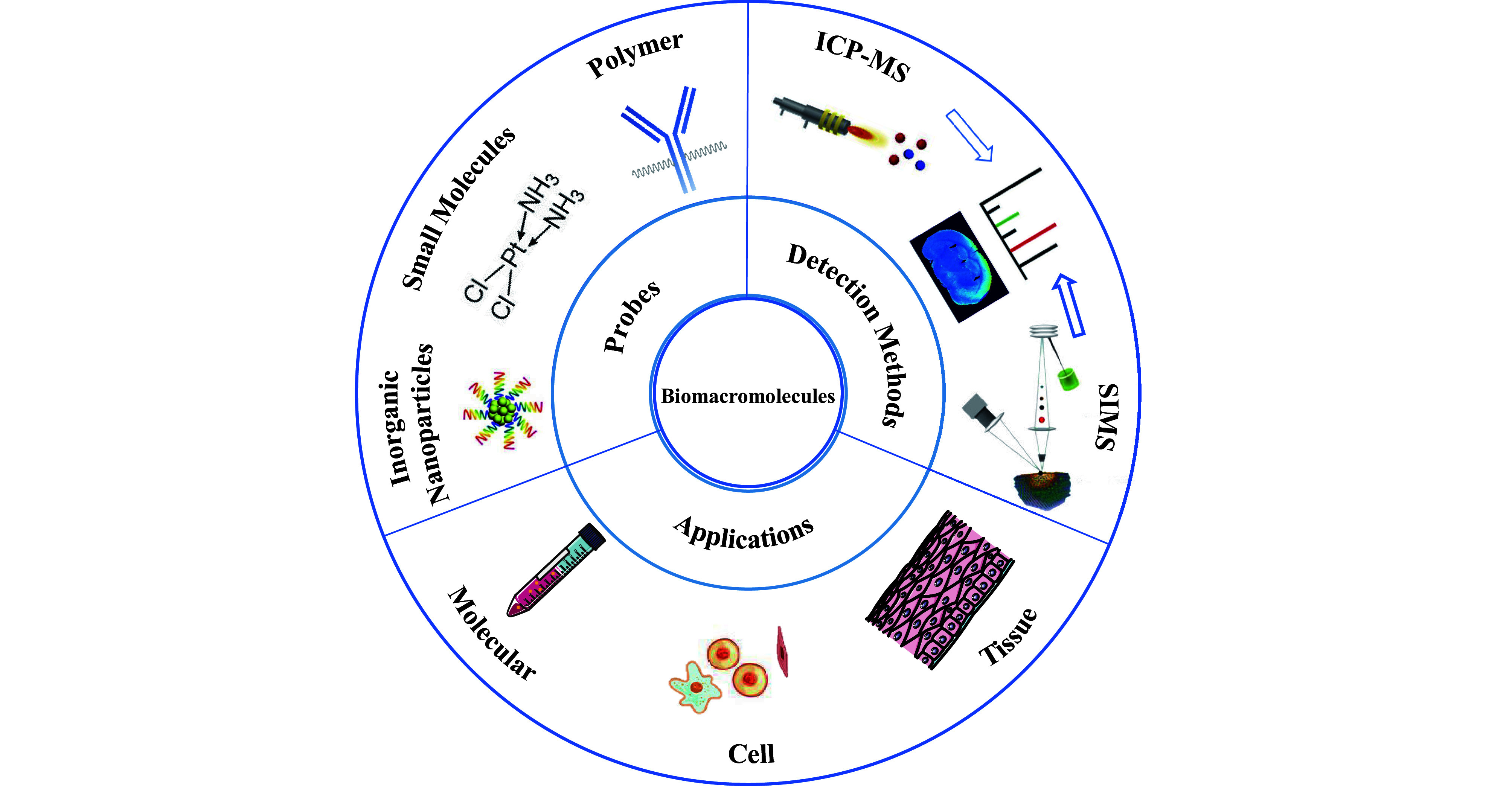
The applications of elemental mass spectrometry techniques combined with metal stable isotopes labeling for the quantification of biomolecules at molecular, cellular, and tissue levels are outlined

This review provides a concise introduction of metal stable isotope labeling combined with elemental MS techniques for biomacromolecules analysis (Fig. 1). First, the constitution and feasibility of exogenous metal stable isotopes probes, normally classified into polymer, small molecule, and inorganic nanoparticle probes, are introduced. Next, we review the current instrumentation state of elemental MS, including ICP-MS and SIMS with the merits of high sensitivity and accuracy, as well as multi-element simultaneous detection ability in the quantification of biomolecules with metal stable isotope labeling. Furthermore, we highlight the practical applications of the combined strategy in biomarkers discovery, clinical pathological analysis, and precision medicine development at molecular, cellular, and tissue levels. Lastly, we conclude with current challenges and prospects for the future directions of the field.

## METAL STABLE ISOTOPE LABELED PROBES

Initially, endogenous non-metal atoms such as sulfur, phosphorus, and selenium were employed for quantifying biomacromolecules as endogenous elemental tags. However, their insufficient detection sensitivity is due to the low elemental ionization efficiency or interference from multi-atomic spectra (He *et al*. 2013). To effectively detect low-abundance biomolecules, exogenous metal stable isotopes are utilized as the key component of the labeling agents. These metal stable isotope labeled probes, which act as reporters can be normally categorized into three groups, including polymer-based probes, small molecule probes, and inorganic nanoparticle probes, *etc*.

### Polymer-based probes

Metal chelating polymers (MCPs) typically incorporate functional groups such as 1,4,7,10-tetraazacyclododecane-1,4,7,10-tetraacetic acid (DOTA) or diethylenetriaminepentaacetic acid (DTPA) to effectively chelate metal stable isotopes as probes (Kumar *et al*. 2023; Schwarz *et* *al*. 2014). These chelators form stable complexes with lanthanide (Ln) metal ions and exhibit low dissociation constants (Han *et al*. 2018). For cell labeling, minimal isotopic exchange occurs between different Ln metal conjugates, allowing the simultaneous coupling of multiple Ln metals to polymers. Initially introduced by Canadian company DVS Sciences by the name Maxpar reagents, MCP reagents are currently marketed by Standard BioTools Inc., which is formerly Fluidigm Inc.

In the meantime, Maxpar X8 Antibody Labeling Kit was developed by Standard BioTools as a commercially available metal isotope labeled probe that utilizes the thiol-maleimide coupling to covalently link MCP with antibodies containing disulfide bonds, such as immunoglobulin G (IgGs). The disulfide bonds in the antibody hinge region are reduced by tris(2-carboxyethyl) phosphine (TCEP) to generate thiols, which then selectively react with maleimide-functionalized MCPs through Michael addition (Arnett *et al*. 2023; Lou *et al*. 2007). Although this bioconjugation has some advantages, it poses challenges for other antibody types like IgMs, IgEs, and IgAs. To overcome this limitation, copper-free click chemistry has been introduced as an innovative conjugation strategy (Allo *et al*. 2018). Terminal azides are enrolled to functionalize MCPs and selectively react with cyclooctyne-functionalized antibodies. The orthogonal chemistry broadens the range of antibody classes and is compatible with the thiol-maleimide conjugation method for preparing bispecific probes.

MCPs covalently conjugated antibodies offer numerous advantages, rendering them an optimal choice for metal stable isotope labeled probes. Nevertheless, the probes necessitate further refinement. Firstly, the available range of metal stable isotopes is limited. Currently, chelating agents such as DTPA and DOTA employed in MCPs preparation predominantly chelate Ln metals (Cho *et al*. 2019), thereby impeding the application of other elements. Therefore, the introduction of novel chelating agents would expand the range of isotopes accessible and empower MCPs-antibodies for multiparametric detection (Zhang *et al*. 2022a; Zhang *et al*. 2022b). Meanwhile, both the capacity for polymers to chelate isotope ions and the potential for antibodies to couple with polymers require enhancement. So far, MCPs can bind 20–50 metal ions per polymer, resulting in chelating approximately 100–250 metal ions per antibody. This quantity is sufficient to detect and quantify highly abundant biomarkers, approximately 10^4^−10^7^ per cell. The highly branched polymers with abundant chelating groups would facilitate greater incorporation of metal atoms, significantly augmenting the analytical sensitivity and accuracy for low abundance biomolecules.

### Small molecule probes

Metal chelates belong to a class of non-polymeric reagents, that utilize functional groups to chelate metal ions (Delgado-Gonzalez and Sanchez-Martin 2021). Apart from Ln elements, metal chelates can incorporate other metal stable isotopes. For instance, new barcode reagents have been developed by chelating six isotopes of palladium through a bifunctional molecule known as isothiocyanobenzyl-EDTA (Zunder *et al*. 2015). These reagents expand the number of measurement channels available for MS and avoid interference with the Ln metal-antibody as labels for measurement.

The DNA metal intercalator, as another small molecule probe can label double-stranded DNA molecules through electrostatic attraction, groove binding, and insertion between nucleotide base units (Ornatsky *et al*. 2008). It is a complex comprising transition metals such as Ir and Rh, two non-intercalating ligands, and one intercalating ligand (Liu and Sadler 2011; Rana *et al*. 2021). In cells stained with DNA metal intercalator, the metal signal is associated with the nucleus. It is therefore most commonly used to identify and count cells. Cisplatin, as a specific DNA metal intercalator, is a well-established chemotherapy drug targeting DNA, which can penetrate non-living cells and form platinum-sulfur bonds with protein through nucleophilic groups (*e*.*g*., R–SH and R–S–R). This property renders it suitable as a cell viability probe (Devine *et al*. 2021). Furthermore, cisplatin can directly label proteins by conjugation with antibodies (Mei *et al*. 2016). Antibodies labeled with cisplatin are compatible with MCPs-antibodies in labeling and washing procedures, without any overlap of isotopic signals in MS detection.

### Inorganic nanoparticles probes

The typical inorganic nanoparticles as MS probes comprise metal nanoparticles, metal nanoclusters (MNCs), quantum dots, metal oxide nanoparticles, upconversion nanoparticles, and metal-organic framework nanoparticles (MOFs). Thereinto, metal nanoparticles consist of densely packed metal atoms, enabling highly sensitive detection of biomolecules (Pichaandi *et* *al*. 2019). However, larger particles may lead to spatial hindrance and reduce labeling efficiency despite stronger detection signals. MNCs comprise a concrete number of metal atoms with diameters less than 3 nm. Due to size proximity to Fermi wavelength, MNCs exhibit intriguing properties such as discrete electronic energy levels, chirality, magnetism, fluorescence, *etc*. (Chakraborty and Pradeep 2017; Cifuentes-Rius *et al*. 2021; Han *et al*. 2021). These properties have been successfully applied in the dual-modal detection of biomolecules using a fluorescence microscope and MS (Zhang *et al*. 2018a). Interestingly, size matching effects allow MNCs similar in size to proteins to achieve a 1:1 labeling radio when used for protein labeling (Li *et al*. 2023a). It is noted that, metal nanoparticles show significant advantages in detecting target biomacromolecules with low expression levels due to the larger number of constituent metal atoms. However, in this case, the nonspecific adsorption capacity is significantly enhanced, which inevitably increases the risk of false positive results. In contrast, MNCs can accurately identify the target in a 1:1 ratio with less nonspecific adsorption, thereby greatly reducing the false positive signals. However, due to the limited number of metal atoms in MNCs, the signal amplification effect is relatively less significant.

As another typical nanometer-sized MS probes, quantum dots are luminescent semiconductor (CdSe, CdS, PbSe, *etc*.) particles with unique optoelectronic properties for dual-modal analysis (Sanmartín-Matalobos *et al*. 2022). In addition, metal oxide and upconversion nanoparticles have also been shown as potential metal labeling reagents (Ngamcherdtrakul *et al*. 2019; Pichaandi *et al*. 2017; Zhang *et al*. 2020b). For instance, each 5.7 nm tantalum oxide nanoparticle (TaO_2_ NP) contains approximately 2700 Ta atoms, while each 10 nm NaHoF_4_ nanoparticle contains about 8000 Ho atoms, and they both have much more amounts of atoms than that of MCPs reagent (Pichaandi *et al*. 2017; Zhang *et al*. 2020b). Similarly, nanoscale MOFs can serve as another class of stable isotope probes to achieve accurate readout of metal signals, which provide a new perspective for these ordered pore structures (Chen *et al*. 2022).

## ELEMENTAL MASS SPECTROMETRY TECHNOLOGY APPLIED FOR ANALYZING METAL STABLE ISOTOPES LABELED BIOMACROMOLECULES

Elemental MS, ICP-MS and SIMS have long been recognized as highly sensitive instruments for detecting the abundance and distribution of biomolecules. Both techniques have contributed greatly to the field of elements analysis (Comi *et al*. 2017). Specifically, ICP-MS lacks sensitivity towards light isotopes, whereas SIMS is an alternative suited for the direct detection of lipids and small molecules but faces challenges in specifically detecting biomacromolecules. In the following section, we mainly introduce the instrument composition, working principle, strengths and shortcomings of the above two MS techniques.

### Inductively coupled plasma mass spectrometry

ICP-MS has long been regarded as a reliable and advanced technology in the field of inorganic element analysis, with exceptional characteristics such as high sensitivity, isotope selectivity, and a wide dynamic range (Liu *et al*. 2011; Wang *et al*. 2010). It integrates with chromatography and laser ablation (LA) systems, and is an irreplaceable analytical tool in life sciences, pharmaceutical research, and environmental monitoring. ICP-MS enables quantitative analysis of naturally occurring elements like sulfur, phosphorus, and metal elements to achieve precise detection of biomolecules (Liu *et al*. 2017). By employing exogenous element labeling of biomolecules, background interference can be significantly alleviated. Notably, the utilization of MCPs and nanoparticles as probes for ICP-MS detection has further augmented its analytical sensitivity owing to the signal amplification (Cid-Barrio *et al*. 2018; Liu *et al*. 2016). It is crucial that, for absolute quantification of biomacromolecule through ICP-MS technique, several criteria should be met. (1) The number of metal atoms in the probe must be accurately determined, which is the basis for accurate quantitative analysis. (2) The determination of the recognition ratio is also indispensable, which involves how to accurately correlate the signal intensity with the actual number of metal atoms in the sample. (3) To avoid the influence of non-specific binding or interference, an effective procedure should be enrolled to separate the redundant probe from the labeled complex.

The mass analyzers of ICP-MS encompass quadrupole, sector field, and time-of-flight analyzers. Presently, the major mass spectrometers available on the market are quadrupole mass analyzers. However, quadrupoles belong to sequential mass analyzers and necessitate switching between two *m*/*z* values for dual isotope ratio measurements. Each switch requires a certain stabilization time to adjust the quadrupole voltage, potentially leading to signal loss during the measurement process. In contrast, inductively coupled plasma time-of-flight mass spectrometry (ICP-TOF-MS) can convert the continuous ion stream generated by ICP into discrete ion packets and precisely analyze each ion packet using TOF. Consequently, it enables the simultaneous acquisition of information from nearly the entire periodic table through transient signals (Au *et al*. 2020, 2022; Lockwood *et al*. 2024). Tian *et al* compared the performance of inductively coupled plasma quadrupole mass spectrometry (ICP-Q-MS), ICP-TOF-MS, and multi-collector inductively coupled plasma mass spectrometry (MC-ICP-MS) in dual isotope analysis of individual nanoparticle (NP) and cell (Tian *et al*. 2023). The results demonstrated that ICP-Q-MS exhibited limited accuracy in determining isotopic ratios within a single NP/cell; whereas MC-ICP-MS encountered challenges in identifying events with minimal metal masses in NPs or cells. In contrast, ICP-TOF-MS has the capability to effectively monitor multiple *m*/*z* values concurrently while ensuring accurate assessment of elemental/isotopic relative abundances.

In 2009, Tanner and his colleagues at the University of Toronto pioneered the integration of traditional flow cytometry with ICP-TOF-MS, resulting in the development of mass cytometry (CyTOF) (Bandura *et al*. 2009). Compared with fluorescence-based flow cytometry, CyTOF effectively overcomes the issue of the overlapping of emission spectra from fluorochromes. This breakthrough enables simultaneous measurement over 40 parameters on individual cell (Bjornson *et al*. 2013; Labib and Kelley 2020; Xie and Ding 2022). CyTOF has now played an irreplaceable role in biological research such as monitoring cell signaling pathways, immune cell differentiation, and inflammation-induced mechanisms (Forbester *et al*. 2020; Herring *et al*. 2018; Krieg *et al*. 2018).

Meanwhile, with the continuous advancements of spatial omics, clarifying the spatial distribution of target biomacromolecules within cells or tissues is imperative. To conquer the dilemma, in 1994, Wang *et al*. pioneered the combination of LA with ICP-MS for bioimaging (Wang *et al*. 1994). In 2005, Hutchinson *et al*. successfully resolved the distribution of β-amyloid protein in Alzheimer’s disease plaques by combining LA-ICP-MS and immunohistochemistry (IHC) for biomolecular imaging analysis (Hutchinson *et al*. 2005). Subsequently, various metal stable isotope labels and multiplexing methods have been developed to enable simultaneous analysis of multiple biomolecules in tissues. Imaging mass cytometry (IMC) technology integrates kinds of metal stable isotopes antibody labeling for LA-ICP-MS imaging, with high spatial resolution and low sample consumption (Yu *et al*. 2020; Zhou *et al*. 2021). It provides a novel method for cell mapping and identification of rare cellular subpopulations. Compared to previous single-event research methods, IMC shows significant potential in pathological research by distinguishing between healthy cells and diseased cells by comprehensively describing and understanding their biological characteristics. Notably, LA-ICP-MS requires cells to be mounted on a substrate, allowing for nearly 100% of individual cells available for analysis. In contrast, a significant number of cells are lost during the sample treatment and instrument processing in CyTOF, resulting in fewer than 50% of cells available for analysis. Additionally, LA-ICP-MS has the advantage of being able to analyze biomarkers from single cells spread across the substrate as well as those distributed within tissue samples.

### Secondary ion mass spectrometry

SIMS was developed in 1960, with the principle of bombarding a sample’s surface using a high-energy primary ion beam. This process induces the absorption of energy and sputtering of surface molecules, generating detectable secondary ions (Boxer *et al*. 2009). SIMS enables the characterization of component and structural features on the sample’s surface. By separating secondary ions with different mass-to-charge ratios through a mass analyzer, an intensity versus mass-to-charge ratio relationship curve can be obtained by counting their respective intensities. This technique offers several advantages, that is, it achieves nanometer-level spatial resolution for precise analysis of micrometer-scale surface areas and captures signals from multiple elements, providing insights into chemical bonding and molecular structures on the sample’s surface (Jiang *et al*. 2016).

From the very beginning, SIMS is mainly used to characterize inorganic materials. Following the invention of static SIMS in 1968, its application has been expanded to encompass organic compound and biomolecule analysis. The integration of TOF into static SIMS during the 1990s propelled TOF-SIMS for investigating biomolecules and associated materials. Concurrently, researchers introduced a novel ion beam instrument tailored specifically for dynamic SIMS applications. This innovation was subsequently commercialized by CAMECA, Inc. by the name NanoSIMS. Despite both dynamic and static SIMS relying on secondary ion detection methods for sample analysis, each technique possesses distinct characteristics (Keren *et al*. 2019; Nuñez *et al*. 2017). NanoSIMS utilizes an energetic primary continuous ion beam, resulting in enhanced sensitivity and spatial resolution. On the other hand, TOF-SIMS employs a pulsed primary ion beam to realize two-dimensional imaging and depth analysis for determining the elemental and molecular composition of samples.

SIMS is also suitable for non-labeled imaging of lipid and metabolite distributions, facilitating direct analysis of the distribution and therapeutic effects of small molecule drugs, metal-containing drugs or biomaterials. Through labeling strategies, SIMS can extend its range of applications. For instance, natural isotopes can be replaced with rare isotopes such as ^13^C and ^15^N, which are then incorporated into metabolic intermediates, enabling the tracking and localization of biological synthesis processes (Baboo *et al*. 2014). However, a limitation of SIMS is its difficulty in detecting specific proteins. Exogenous labeling is thus more suitable for detecting proteins with larger molecular weight. Although fluorine-19 (^19^F) has been utilized as a protein label (Vreja *et al*. 2015), its high background signal complicates the detection of biomacromolecules with low abundance (Agüi-Gonzalez *et al*. 2021). In contrast, the probes comprising noble metals and Ln metals are scarce in biological samples and easily ionizable. Hence, metal stable isotope probes are well-suited for quantitative analysis and imaging of biomacromolecules using SIMS (Jin *et al*. 2022; Li *et al*. 2022).

As another rapid technological advancement of SIMS in recent years, the combination of multiple ion beam imaging (MIBI) and metal stable isotope labeling has found widespread application. By simultaneously quantifying dozens or even hundreds of proteins or RNAs, it improves the comprehensive spatial characterization of cellular subtypes. MIBI is particularly well-suited for evaluating intracellular biomolecules and elucidating cell phenotypes, epigenetics, and metabolic states. The high-definition multiple ion beam imaging (HD-MIBI) developed in 2021 achieves an impressive lateral resolution as low as approximately 30 nm (Rovira-Clavé *et al*. 2021), rendering this technique potentially indispensable for subcellular resolution analysis of biomolecules. In addition, multiplexed beam imaging by the time of flight (MIBI-TOF) is frequently employed to enable the simultaneous detection of numerous proteins, which facilitates the quantitative analysis, distribution visualization, and spatial proteomics research following the elemental mass tags staining (Keren *et al*. 2019; McCaffrey *et al*. 2022; Risom *et al*. 2022).

## APPLICATIONS OF METAL STABLE ISOTOPES LABELING COUPLED WITH ICP-MS AND SIMS FOR BIOMACROMOLECULES ANALYSIS

Using metal stable isotope labeling with MS technology allows for analysis of almost all biological analytes, representing a significant advancement that offers a more comprehensive and precise analytical approach. In the following section, we will emphasize the typical applications of ICP-MS and SIMS in analyzing biomacromolecules at molecular, cellular and tissue levels by different detection methods, respectively. Table 1 summarizes the recent reports that metal stable isotopes labeling to analyze expression levels and spatial distribution of biomolecules across various levels.

**Table 1:** Typical examples of applications of metal stable isotopes labeling-assisted biomacromolecules analysis at different levels

Analytical level	Detection method	Sample	Sample processing method	Elements or metal stable isotopes for analysis	Analytes and the relevant biological issues	References
Molecular level	ICP-MS	Serum	-	Au	Measurement of alpha-fetoprotein concentration	Li *et al*. 2018
	ICP-MS	Serum	-	Tb, Ho and Lu	Detection of miRNA-21, miRNA-155, and miRNA-10b	Kang *et al*. 2021
	SP-ICP-MS	Serum	-	Au, Ag and Pt	Non-wash heterogeneous immunoassay for gastric cancer biomarkers CA724, CA199, and CEA	Huang *et al*. 2022
Cellular level	CyTOF	Peripheral blood	-	Ln	Classification of the severity of COVID-19 patients through analysis of TMPRSS2, CD163/CD206, and CD33	Martínez-Diz *et al*. 2022
	CyTOF	MCF-7, Ramos, DC2.4, B16F10 and mouse splenocytes	-	Zr isotope	CD45 detection at single-cell level	Dang *et al*. 2021
	CyTOF	Peripheral blood	-	^198^Pt	Collection and phenotypic detection of cells in trace amounts of PBMC	Li *et al*. 2023b
	SC-ICP-TOF-MS	Yeast strains and algae	-	Ru	Cell volume determination	Qin *et al*. 2021
	SC-ICP-TOF-MS	Arpe-19 cells	-	Ir, Pt, Au and Ru	Measurement of relative concentrations of Hepcidin, MT-2, and Ferroportin within individual cells	Menero-Valdés *et al*. 2023
	ICP-MS	Blood sample	-	Au	Recognition and capture of individual CTC	Zhang *et al*. 2021
	ICP-MS	Blood sample	-	Tb	CTCs capture for early diagnosis and treatment of cancer	Yin *et al*. 2020
	LA-ICP-TOF-MS	THP-1 cells	-	Ho, Ir	Measurement of the changes in endogenous isotope content throughout the entire cell cycle	Löhr *et al*. 2019
Tissue level	IMC	Pancreatic ductal adenocarcinoma tissue in mice	Frozen tissue sections	Ln	The spatial distribution of cells and their interaction in TME	Erreni *et al*. 2024
	IMC	Colon tumor and tonsil tissue of humans	Formalin-fixed paraffin-embedded tissue sections	Ln, Cd, Pt, and Y	Interaction between cancer cells and immune cells in the cancer-immune microenvironment.	Ijsselsteijn *et al*. 2019
	MIBI	Human brain tissue	Formalin-fixed paraffin-embedded tissue sections	Ln	Investigation of brain cells, tissue architecture, and phenotypic variations across different stages of Alzheimer’s disease	Vijayaragavan *et al*. 2022
	MIBI	Himalayan monkey lymphoid tissue	Formalin-fixed paraffin-embedded tissue sections	Ln	Synergistic immune events in the lymphoid tissue reservoirs of retroviral infection	Jiang *et al*. 2022
	MIBI	Small cell lung cancer xenotransplantation model tissue from mice	Formalin-fixed paraffin-embedded tissue sections	Pd isotope	Spatial distribution of cellular phenotypes in tissue derived from a small cell lung cancer xenograft model	Rovira-Clavé *et al*. 2022
	IMC	Prostate cancer tissue	Formalin-fixed paraffin-embedded tissue sections	Ln	Detecting PSMA, NCL and EpCAM for prostatic adenocarcinoma diagnosis	Yu *et al*. 2021
	LA-ICP-TOF-MS	Mouse brain tissue	Frozen tissue sections	Au	Precise diagnosis of early Alzheimer’s disease through analyzing connective tissue growth factor expression between AD patients and healthy individuals	Lu *et al*. 2024

### Molecular level

**Figure 2:**
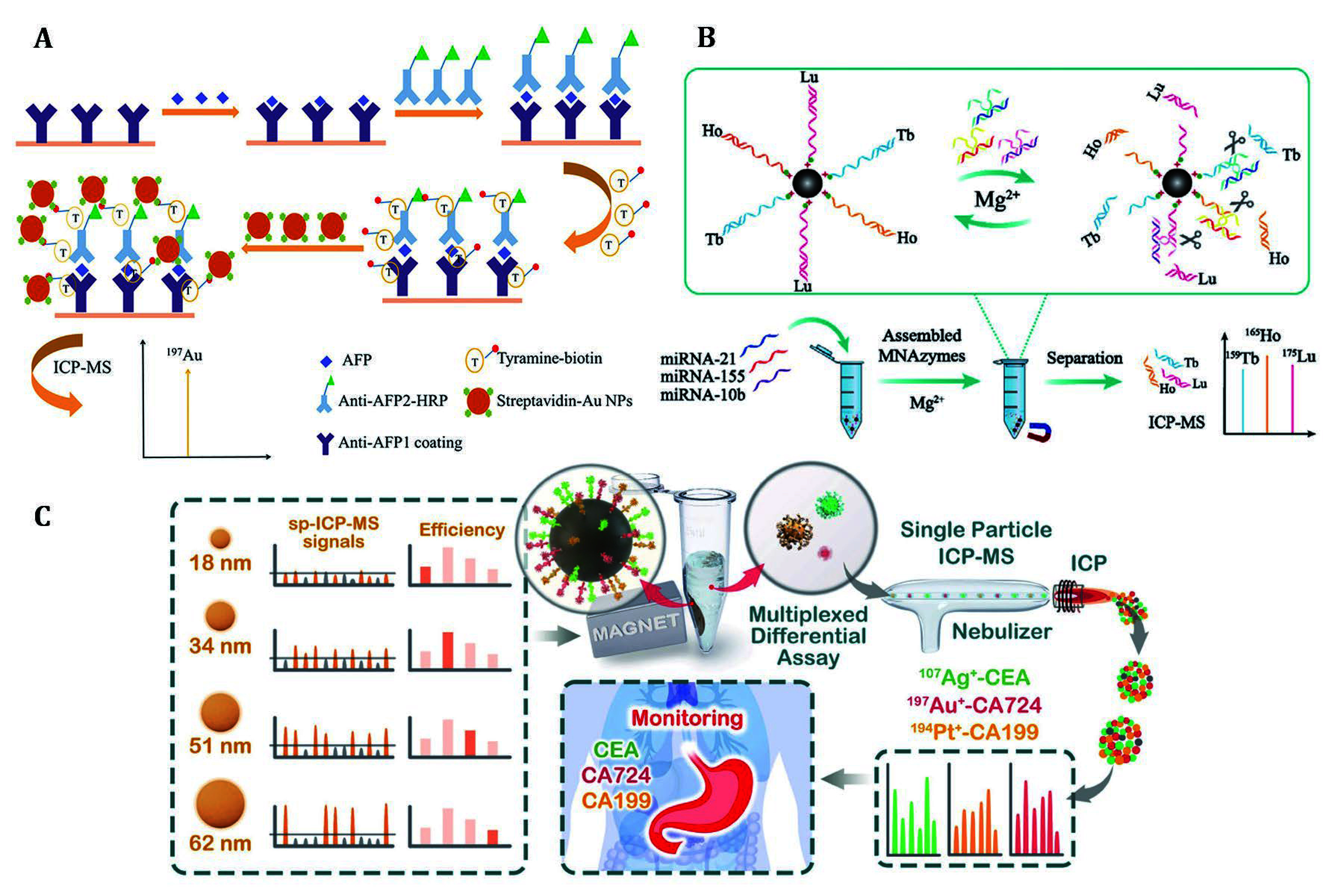
Enhanced detection sensitivity of free protein and miRNA through amplification strategies. **A** Free AFP was evaluated by the amplified elemental signal that was obtained by catalyzing the deposition of biotinylated tyramine and coupling with gold nanoparticles (Li *et al*. 2018). **B** The MNAzyme amplification strategy was combined with Ln elements labeling to enable sensitive detection of three miRNAs (Kang *et al*. 2021). **C** Three tumor biomarkers in the biological fluid were determined by evaluating the concentration of metal nanoparticles before and after immunoreaction with magnetic microspheres using SP-ICP-MS (Huang *et al*. 2022)

The first work for quantifying biomacromolecules at the molecular level by specific metal probe labelling was reported by Zhang and co-workers for the assessment of thyroid-stimulating hormone (TSH) in human serum (Zhang *et al*. 2001). Since then, the use of Ln consists of 14 stable elements as reporters allowing for highly multiplexing biomolecule analysis with good sensitivity and precision in mixed biological samples (Tholey and Schaumlöffel 2010). To achieve higher analytical sensitivity, amplification is a preferred choice. In 2018, as shown in Fig. 2A, Hu and his colleagues pioneered the integration of tyramine signal amplification with the self-amplification effect of gold nanoparticles (Li *et al*. 2018). Through this dual amplification strategy combined with ICP-MS technology, they successfully detected alpha-fetoprotein (AFP) in serum with an impressive detection limit of 1.85 pg/mL. Furthermore, for miRNA detection, they developed an MNAzymes amplification strategy using streptavidin-modified magnetic beads and three Ln-labeled (^159^Tb/^165^Ho/^175^Lu) single-stranded DNA substrates (Kang *et al*. 2021). As shown in Fig. 2B, three types of MNAzymes hybridize with their respective target miRNAs, which induces the cleavage and release of Ln-labeled substrates. The cleaved MNAzymes then cyclically cleave another substrate on the probe to achieve signal amplification.

Single nanoparticle analysis is an alternative method to enhance sensitivity. An advantage of single particle inductively coupled plasma mass spectrometry (SP-ICP-MS) is the detected signal pulse corresponds to a single NP, enabling rapidly determining the size, size distribution, and concentration of metal NPs in suspension. SP-ICP-MS was successfully employed for assessing serum biomarkers CA724, CA199, and CEA of gastric cancer patients by Huang *et al*. (Huang *et al*. 2022). As shown in Fig. 2C, firstly, noble metal NPs, antibody-labeled magnetic microspheres and target proteins were mixed. Then noble metal NPs bound to the target proteins on the surface of magnetic microspheres through sandwich immuno-recognition. Subsequently, magnetic microspheres were separated magnetically, and the unreacted metal NPs were collected and quantified to assess the concentration of the target proteins. It provides an accurate and convenient method for monitoring malignant tumor prognosis and recurrence, especially when combined with biomarker panel strategies.

### Cellular level

**Figure 3:**
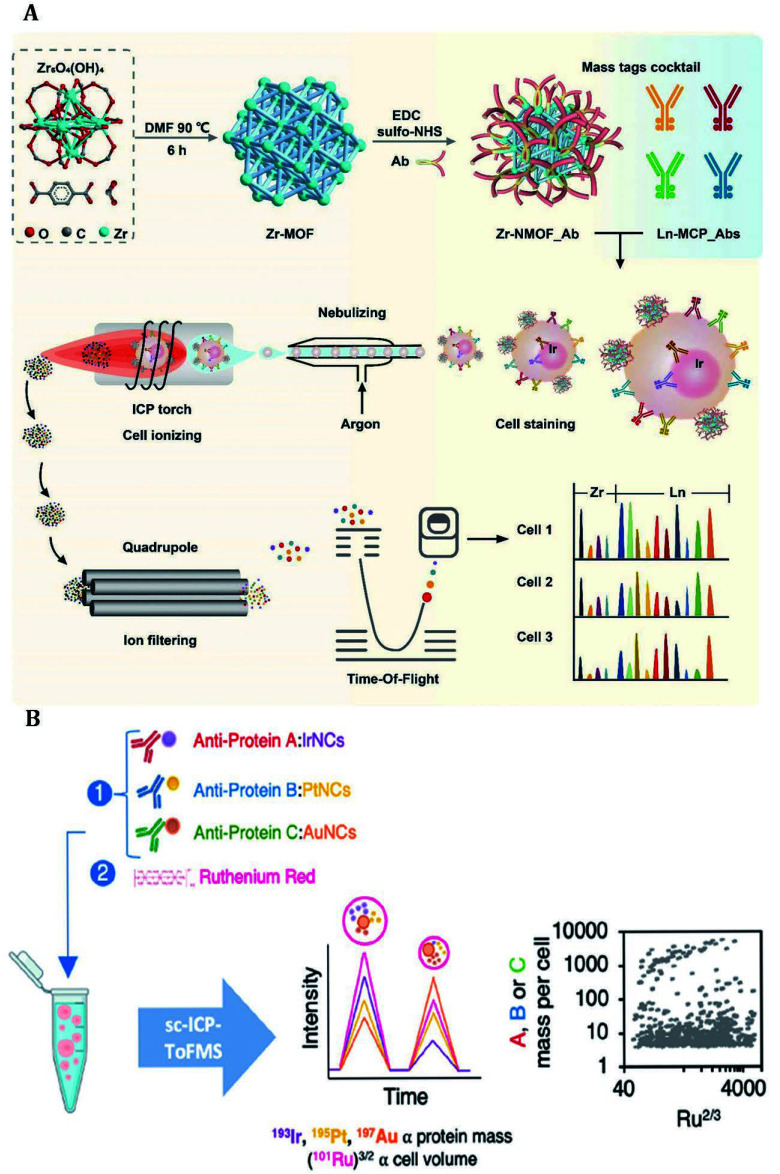
**A** Zr-NMOF was synthesized and bioconjugated with antibodies by EDC/sulfo-NHS reaction. Zr-NMOF targeting CD45, together with MCP-Abs targeting CD19, CD3, CD4, and CD8 were used for cell staining, while DNA labeling was accomplished through iridium insertion. High-temperature plasma evaporation of the samples generated element ion clouds for identification and qualitative analysis of cellular biomarkers by TOF-MS (Dang *et al*. 2021). **B** By labeling ferritin, metallothionein-2, and transferrin with antibodies decorated metal clusters, the relative concentration of proteins in individual cells was directly accessed by using RR staining to determine individual cell volume (Menero-Valdés *et al*. 2023)

Cells are the most important research object for revealing the mechanism of disease development and exploring therapeutic approaches. The Maxpar commercial assay kits have been extensively employed in the investigation of various cellular biomarkers. The assay kits typically incorporate Ir-DNA intercalators for nucleic acid labeling, cisplatin for discriminating between live and dead cells, or MCPs conjugated with antibodies for specific biomacromolecule targeting. For instance, Martínez-Diz *et al*. utilized the Maxpar Human Monocyte/Macrophage Phenotyping Panel Kit to access peripheral blood samples of COVID-19 patients by CyTOF (Martínez-Diz *et al*. 2022). By using a panel comprising 15 Ln metal probes alongside TaqMan probes for genotyping, they correlated several markers such as TMPRSS2, CD45^-^, CD163/CD206, and CD33 with COVID-19 invasiveness, providing evidence for patient classification in a clinical setting. Similarly, Maxpar commercial assay kits have also found applications in research areas including immunotherapy and the investigation of mechanisms underlying the onset complications (Krieg *et al*. 2018; Shen *et al*. 2022). However, as shown in Fig. 3A, owing to the limited availability of Ln isotopes, Ding and colleagues developed antibody-modified Zr-NMOFs compatible with four additional detection channels alongside MCPs-antibodies to enable simultaneous labeling of multiple proteins (Dang *et al*. 2021). They also devised ^198^Pt-cisplatin that can rapidly penetrate the damaged cells and non-specifically interact with proteins, peptides, and amino acids to label carrier cells (Li *et al*. 2023b). The inclusion of carrier cells boosts initial counts during the detection process which helps to counteract the reduced resolution and distorted analysis results when examining target cells. This approach is congruent with viability assays based on cisplatin.

Single-cell analysis uniquely identifies and characterizes cellular heterogeneity. Additionally, different from the targets distributed on the cytomembrane, accurately assessing the cell volume is crucial for determining the quantity of the intracellular analytes in individual cell. Recently, Qin *et al*. proposed a novel approach for precise determination of cell volume by detecting ruthenium red (RR) binding with polysaccharides on the cell surface by single-cell (SC)-ICP-TOF-MS analysis (Qin *et al*. 2021). The method exhibited species-dependent staining intensity and enabled estimation of cellular volume when cells exhibit regular morphology. Further, as present in Fig. 3B, Menero-Valdés *et al*. employed RR along with three metal clusters to label suspended cells, investigating both the quantity and relative concentration variations of ferritin, metallothionein-2, and transferrin within each cell under two distinct stress conditions (Menero-Valdés *et al*. 2023). These findings provide valuable insights into studying cellular heterogeneity and offer a deeper comprehension of biochemical processes at an individual cellular level.

**Figure 4:**
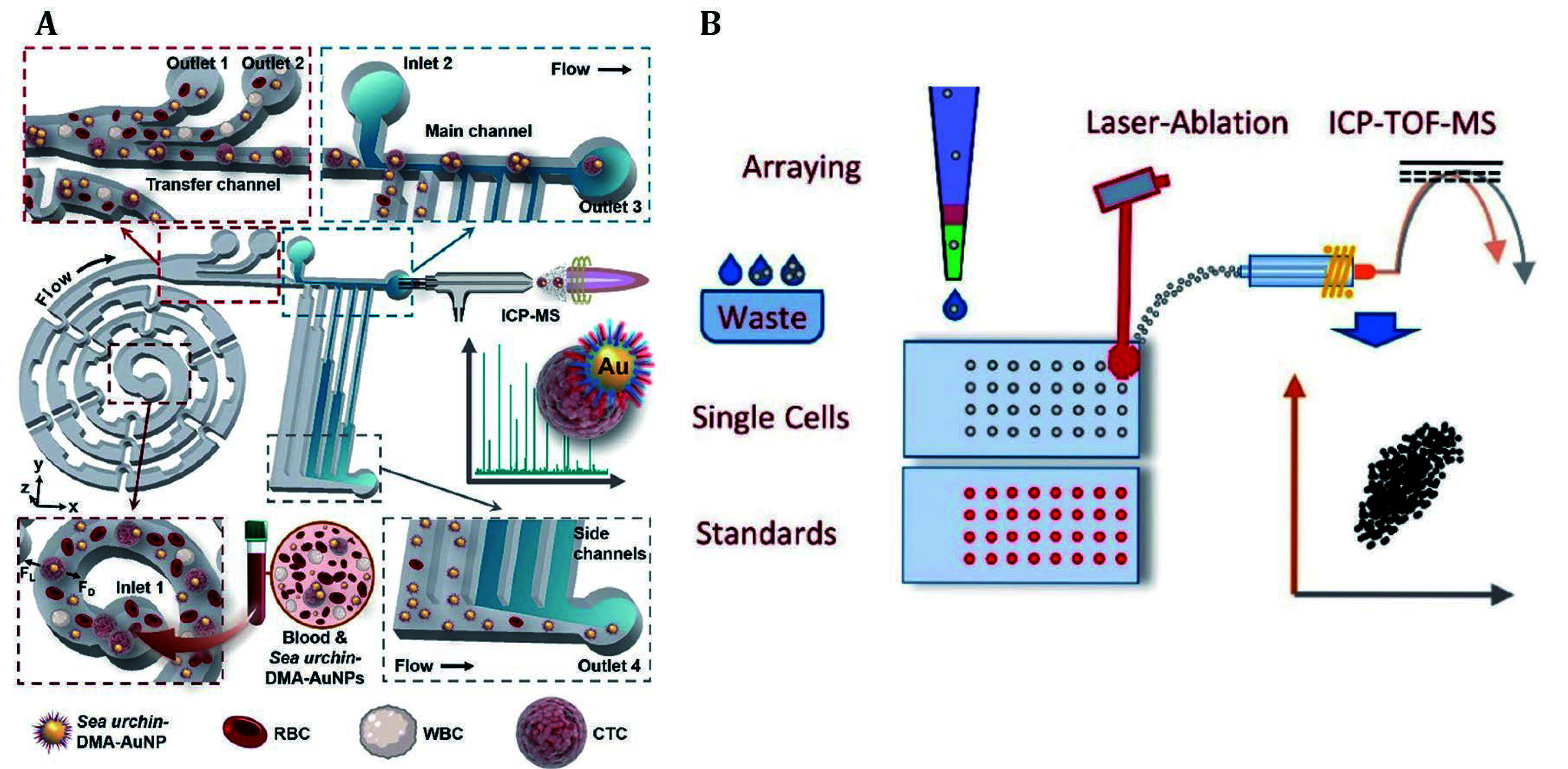
**A** The sea urchin-inspired single-cell CTC recognition platform was developed, which captured CTCs by incubating sea urchin-DMA-AuNPs with blood. The CTCs were then separated from other cells and free probes in a chip based on secondary flow and hydrodynamic filtration. Subsequently, individual CTCs were subjected to background-free single-cell analysis by time-resolved ICP-MS (Zhang *et al*. 2021). **B** The pressure-acoustic single-cell microarray was developed for the detection of endogenous and exogenous isotopes within cells by LA-ICP-TOF-MS (Löhr *et al*. 2019)

Circulating tumor cells (CTCs) are rare tumor cells that are released from primary tumors and circulate in the bloodstream for remote metastasis, which act as prognostic indicators for breast, prostate, colorectal cancer and other cancers (Lawrence *et al*. 2023; Nikanjam *et al*. 2022; Sarioglu *et al*. 2015). Zhang *et al*. developed a sea urchin-inspired single CTC recognition platform (Zhang *et al*. 2021). As illustrated in Fig. 4A, this platform integrated a dual aptamer connected to AuNPs (sea urchin-DMA-AuNPs) that facilitated efficient capture and sorting of CTCs, and utilized ICP-MS for background-free analysis. This innovative platform demonstrated a remarkable 100% CTC capture efficiency, enabling rapid detection of individual CTCs within just one hour from a mere 100 μL of whole blood.

To further achieve non-destructive counting, release and culture of CTCs, Yin *et al*. developed a multifunctional platform. This platform effectively captured MCF-7 cells by recognition of mucin 1 (MUC1) on the cell membrane with aptamer-decorated capture probes (Yin *et al*. 2020). Subsequently, the starting primer hybridized with the aptamer was replaced by MUC1 and released into solution. The released primer was separated from the captured cells and hybridized with Tb-labeled substrate on detection probes. This process was followed by releasing a large amount of nicked Tb fragments through the nicking endonuclease-assisted amplification, enabling subsequent ICP-MS detection. On the other hand, after digestion by nucleases, the captured cells were detached from the probe and were continuously cultured. Finally, ICP-MS counting demonstrated a recovery rate of 52.7% at a detection limit of 87 for MCF-7 cells, while maintaining a cellular viability of 74.3%. Moreover, the demand for faster single-cell detection has driven advancements in the ICP-TOF-MS technique toward higher speed. As present in Fig. 4B, Löhr *et al*. developed a single-cell array and integrated it with LA-ICP-TOF-MS for quantitative analysis and isotopic fingerprinting, achieving high-throughput detection of 550 cells per hour (Löhr *et al*. 2019). In this work, the precise operation and arrangement of single cells have been successfully achieved by the single-cell array. In detail, Ir-DNA and mDOTA-Ho were used to stain the DNA and protein of the suspension cells, respectively. With the aid of aligned droplets containing the same probe at known concentrations and the LA-ICP-TOF-MS technique, both probes can be quantified at the single-cell level. In addition, this work examined the isotopic fingerprint of single cells, and employed Ir-DNA as a biomarker to assess cell cycle status. It was found that Zn and P contents increased as the cell period moved from G1 to S and G2. This indicates that elemental biomarkers are related to changes in the content of endogenous isotopes.

### Tissue level

Conventional histopathologic analysis methods, such as immunofluorescence, primarily employ fluorescently labeled antibodies for tissue section staining and subsequent microscopic examination to assess the distribution of target analytes within the tissue. However, this method allows for the analysis of a limited number of biomarkers, it becomes valueless when dealing with an increased number of biomarkers due to spectral overlap issues (Angelo *et al*. 2014). Alternatively, metalstable isotope barcoding circumvents spontaneous fluorescence interference, thereby reducing background signals. In a typical process, histopathologic tissues are primarily acquired through fresh freezing, formalin fixation, or paraffin embedding. Then metal stable isotope-coded probes label the multiplexed biomarkers expressed on tissues. Lastly, high-resolution MS techniques enable simultaneous detection of numerous metal isotopes labeled-antibodies recognized biomacromolecules.

IMC refers to a technology that ablates tissue sections using a laser, brings the ablated component through an inert gas flow into an inductively coupled plasma ion source, and performs isotopic analysis via a mass flow cytometer with a TOF mass analyzer. Recently, Erreni *et al*. developed a panel of 28 markers specifically designed for detecting the spatial distribution, as well as the interactions of tumor cells and immunocytes in the tumor microenvironment (TME) at the frozen tissue sections level (Erreni *et al*. 2024). This panel enables effective cell segmentation and phenotypic clustering, facilitating IMC analysis of TME cell distribution in different pancreatic ductal adenocarcinoma (PDAC) mouse models. Moreover, additional markers can be incorporated to expand this panel, unraveling the complexity of PDAC and providing insights into prognosis or treatment options for this disease.

As another representative study, Ijsselsteijn *et al*. developed a panel of 40 markers for determining major immune cell subsets by IMC, as well as investigating tumor-immune cell interactions within the tumor-immune microenvironment on formalin-fixed paraffin-embedded (FFPE) tissues (Ijsselsteijn *et al*. 2019). To ensure full depth penetration of the probes into the tissue, the antibodies were allowed for precise control over incubation temperature and duration time to maximize the antibodies’ performance, while maintaining signal intensity and specificity for antigen detection.

MIBI-TOF MS has a higher spatial resolution than ICP-TOF-MS down to 250 nm, and is therefore used for high-resolution elemental analysis of tissues. Vijayaragavan *et al*. validated 39 antibodies to identify brain tissue-specific targets through conventional IHC, the targets were then labeled and imaged with MIBI-TOF MS (Vijayaragavan *et al*. 2022). Human brain FFPE samples were stained with 36 distinct metal isotopes labeled antibodies, followed by images of various brain regions at different stages of Alzheimer’s disease using a 36-dimensional MIBI-TOF model. This method enabled the identification of cell types and protein pathologies associated with diverse stages of Alzheimer’s disease in the hippocampus.

**Figure 5:**
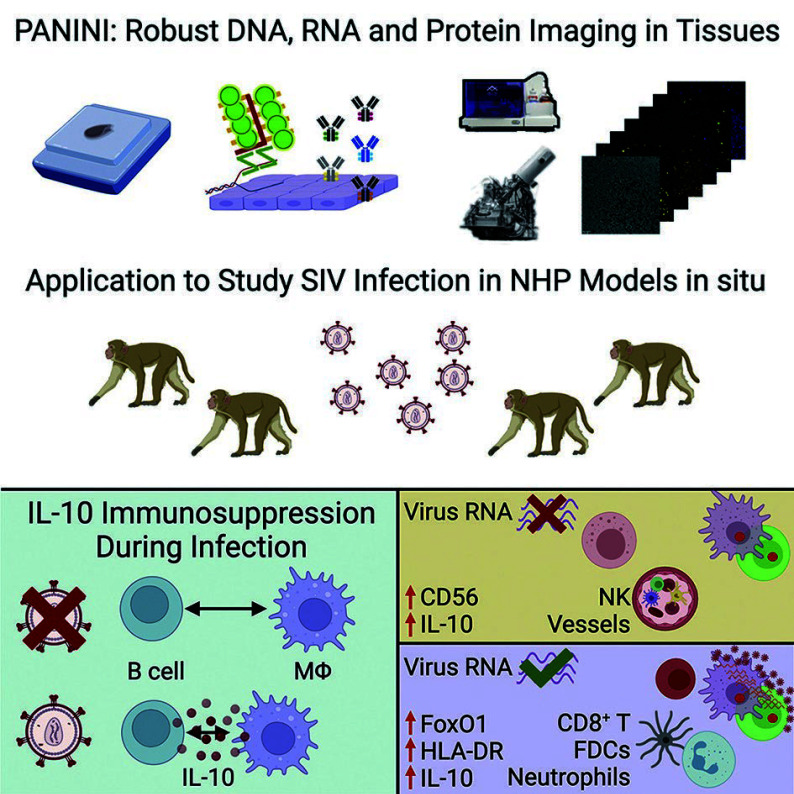
The protocol began with nucleic acid amplification and peroxidase-catalyzed hapten deposition, followed by the labeling of nucleic acid and protein targets on sliced tissues. Subsequently, multiplexed images were acquired by following MIBI and CODEX platforms, and computational analysis was conducted to elucidate the host-pathogen interactions with high resolution (Jiang *et al*. 2022)

As the pioneer researchers in this field, Nolan and colleagues at Stanford University have developed a robust method known as PANINI, which integrates nucleic acid and protein imaging (Jiang *et al*. 2022). As shown in Fig. 5, this method employs protease-free branched-chain amplification of nucleic acids, tyramide signal amplification combined with hapten deposition for MIBI imaging to simultaneously quantify the expressing levels of viral DNA (vDNA), viral RNA (vRNA), and 31 proteins within tissue sections. It was utilized to analyze viral reservoirs and immune responses in lymphoid tissues from simian immunodeficiency virus SIV-infected and uninfected controls, revealing synergistic immune events in the lymphoid tissue reservoirs of retroviral infection. In the study of viral infection mechanisms, simultaneous analysis of proteins and nucleic acids can determine the state of infected cells and virus-host interactions, and can also reveal the immune regulatory process of SIV-infected tissues.

Additionally, Nolan and his colleagues developed EpicTags, a combined labeling technique using six palladium isotopes that merged with MIBI (EpicMIBI) for in situ tracking of barcodes within the tissue microenvironment (Rovira-Clavé *et al*. 2022). EpicTags enable barcode encoding for 20 cell lines and were employed to dissect spatial components of cell lineages and phenotypes in xenograft models of small cell lung cancer. EpicMIBI facilitates in situ detection of tumor heterogeneity related to both cell-intrinsic and cell-extrinsic processes. Its capability to track barcoded cancer cells in situ, along with their surrounding spatial environment, opens new opportunities for understanding tumor differentiation.

**Figure 6:**
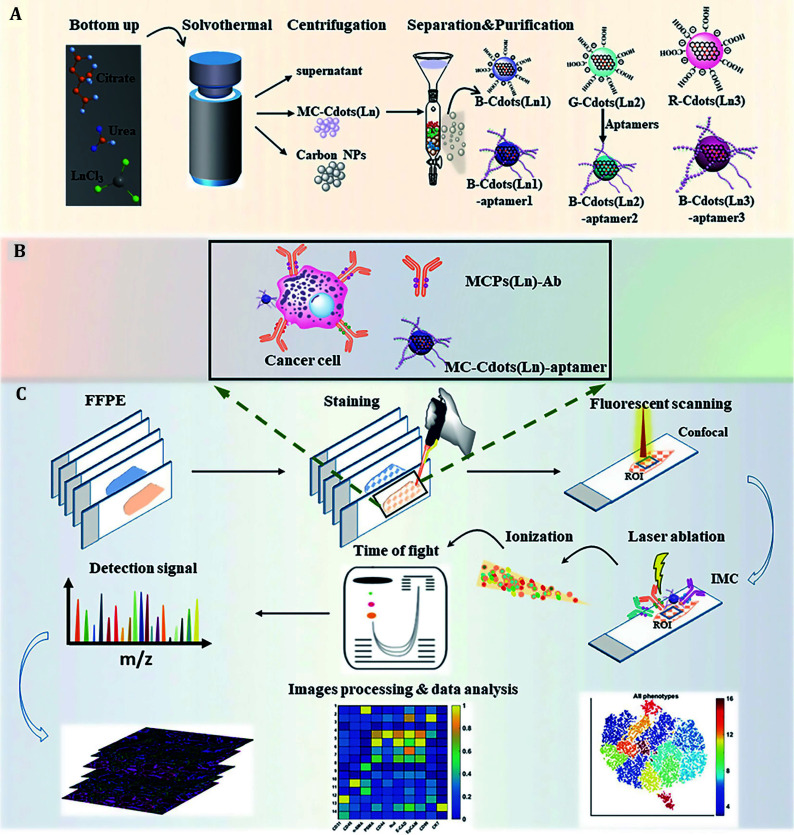
The strategy employed bimodal probes for the rapid identification and detection of ROI, thereby reducing the time of MS scanning. **A** The synthesis and purification of MC-Cdots (Ln) were performed to prepare bimodal probes. **B** A combined library consisting of the aptamer labeled MC-Cdots (Ln) and Maxpar antibody reagents was utilized for sample labeling. **C** MC-Cdots (Ln)-aptamer acted as a bimodal probe for the rapid identification of ROI through fluorescence signal, facilitating multiplex detection of ROI on the same tissue section by IMC (Yu *et al*. 2021)

Despite the notable advantages of MS imaging, its speed has consistently posed a significant challenge, hindering its clinical application. One effective approach to address this issue involves employing fluorescence analysis to identify regions of interest (ROI) initially, followed by subsequent MS analysis. As illustrated in Fig. 6, Yu *et al*. have developed a series of blue, green, and red fluorescent Ln-doped multicolor carbon nanodots (MC-Cdots (Ln)) as dual-mode probes for both fluorescence and mass detection. These nanodots were combined with Maxpar antibody reagents for tissue analysis (Yu *et al*. 2021). By rapidly identifying ROIs by traditional immunofluorescence and subsequent multiplex IMC analysis, this strategy reduces blind scanning time for IMC by 90% while compensating for its low resolution. This methodology offers a promising solution for clinical applications that necessitate rapid imaging.

Further, an “all in one” probe was fabricated to facilitate a multi-modal accurate diagnosis of early-stage Alzheimer’s disease. Recently, Lu *et al*. employed gold NCs that penetrate the blood–brain barrier (BBB) and target connective tissue growth factor (CTGF) expressed in the brain to analyze CTGF in early-stage APP/PS1 transgenic mice (Lu *et al*. 2024). The abnormal overexpression of CTGF predate Aβ deposition could be a potential biomarker of early-stage AD. Moreover, combined with ICP-MS quantitative techniques, through *in vivo* NIR-II imaging, *in vitro* visible fluorescence and peroxidase-like colorimetric imaging, the multi-modal analysis of CTGF on postmortem brain slices was conducted to differentiate AD patients from healthy individuals. In this case, the detection methods of different modalities can complement each other and provide valuable information for a comprehensive understanding of the AD pathological process. With NIR-II imaging, the probe can be well home to the elevated CTGF *in vivo*, enabling noninvasive and real-time early elevation of CTGF. The spatial distribution and overall expression level of CTGF in mouse brain sections can meanwhile be evaluated by fluorescence and peroxidase-like colorimetric imaging. Thereafter, CTGF expression analysis on brain pathological tissue level was performed by LA-ICP-TOF-MS, verifying the consistency of cell analysis and molecular imaging analysis, and finally providing the “gold standard” for diagnosis.

## SUMMARY AND PERSPECTIVES

Biologists heavily rely on detecting biomolecular abundance and distribution to deepen their understanding of life processes. Accurate quantification and clarifying the distribution of biomolecules is crucial for both fundamental biological research and practical clinical diagnostics. Accompanied by the advancements of metal stable isotope labeling and exogenous tags, including polymer, small molecule, and inorganic nanoparticle probes, elemental mass spectrometry instruments have emerged as indispensable tools for analyzing various target biomacromolecules. The review briefly summarizes the applications of ICP-MS and SIMS with elemental tags for quantifying biomacromolecules including proteins and nucleic acids. Particularly in the field of multiparametric biomacromolecule analysis, it outperforms traditional spectroscopic methods regarding sensitivity and resolution. However, we still face many unresolved issues and challenges. Although there are currently nearly 50 isotopes used for biomacromolecule labeling, there are still many non-biological element isotopes that have not been applied. To address these issues, new labeling reagents need to be designed to utilize more elemental isotopes while optimizing the specificity and efficiency of labeling. Additionally, continuous improvement and breakthroughs in detection methodology are also needed to achieve lower detection limits, higher resolution, faster speed, and greater throughput. We anticipate metal stable isotope labeling strategies combined with elemental mass spectrometry technique playing a more confounding role in routine laboratory and clinical settings, leading to a deeper understanding of physiological processes across different fields.

## Conflict of interest

Ping Zhang, Ying Han, Yue Xu and Liang Gao declare that they have no conflict of interest.
